# Augmented RFID Technologies for the Internet of Things and Beyond

**DOI:** 10.3390/s20040987

**Published:** 2020-02-12

**Authors:** Luciano Tarricone, Jasmin Grosinger

**Affiliations:** 1 Department of Engineering for Innovation, University of Salento, 73100 Lecce, Italy; 2 Institute of Microwave and Photonics Engineering, Graz University of Technology, 8010 Graz, Austria; jasmin.grosinger@tugraz.at

**Keywords:** RFID and sensing, embedded sensors, SAW RFID sensors, tag antennas, chipless RFID, localization, security in RFID systems, RFID for IoT

## Abstract

Radio frequency identification (RFID) is one of the crucial enabling technologies for the Internet of Things (IoT). This is leading to a continuous augmentation of RFID technologies, in terms of sensing capabilities, energetic autonomy, usability, and cost affordability, and this special issue proposes an overview on such a challenging scenario. The proposed results, in terms of cost reduction, miniaturization, and compatibility with complex systems and technologies, as well as the identification of the relevant criticalities, also pave the way to future steps being taken that go beyond the current IoT.

## 1. Introduction

With the availability of inexpensive, low-powered integrated circuits since the early 1990s, considerable research and development efforts have been made in the area of radio frequency identification (RFID) systems for identification (ID) purposes. More recently, researchers have been working consistently towards the goal of reaching beyond the ID in RFID, i.e., by integrating sensing capabilities in RFID transponders (tags) to additionally monitor the tag environment, such as temperature, curvature, or liquid level. 

Important results have been achieved in terms of cost reduction, miniaturization, and compatibility with complex systems and technologies. Meanwhile, pervasive and affordable computing and communication technologies have opened challenging scenarios for the Internet of things (IoT) with its manifold implications and constant growth of applications, bringing technology more and more into daily life and even inside living systems. Some crucial issues have emerged in the context of IoT, such as security, connectivity, sustainability, and compatibility with living systems. In this framework, this special issue presents the latest research results and a review on RFID technologies that push the state of the art to go beyond the ID in RFID, to enhance IoT, and to pave the way for future steps.

## 2. Summary of Special Issue Papers

The review article “Radio Frequency Identification and Sensing Techniques and Their Applications—A Review of the State of the Art” [1] presents an overview of sensor tags based on high frequency (HF) RFID, ultra-high frequency (UHF) RFID, and chipless RFID technologies. In particular, the article presents selected work on tag antenna designs and antenna printing techniques and on the RF subsystem design of tag chips and chip products, highlighting applications of sensor tags and technical challenges.

The research paper “Smart RFID Sensors Embedded in Building Structures for Early Damage Detection and Long-Term Monitoring” [2] presents the results of the RFID humidity sensor tags used for structural health monitoring of concrete structures. The paper successfully demonstrates the long-term use of HF and UHF RFID sensor tags based on off-the-shelf components in concrete structures, investigating sensor tag material degradation in time.

The research paper “Multi-Channel Real-Time Condition Monitoring System Based on Wideband Vibration Analysis of Motor Shafts Using SAW RFID Tags Coupled with Sensors” [3] presents surface acoustic wave (SAW) based RFID sensor tags used for wireless vibration monitoring of rotating machine parts. The paper demonstrates successfully the use of SAW based RFID sensor tags based on off-the-shelf components on rotating components, demonstrating real-time vibration measurements for predictive maintenance.

The research paper “Wirelessly Powered Light and Temperature Sensors Facilitated by Electrically Small Omnidirectional and Huygens Dipole Antennas” [4] presents chipless UHF RFID sensors tags for brightness and temperature monitoring. The paper successfully demonstrates two different types of tag antenna types, providing different radiation characteristics for a well-targeted wireless power transfer in the specific application.

The research paper “Longest-Range UHF RFID Sensor Tag Antenna for IoT Applied for Metal and Non-Metal Objects” [5] presents a novel tag antenna design for UHF RFID tags. The paper successfully demonstrates that the specific tag antenna leads to a tag read range of about 26 m in free space, operating equally well in the case of metal or non-metal objects, respectively.

The research paper “Super-Wide Impedance Bandwidth Planar Antenna for Microwave and Millimeter-Wave Applications” [6] proposes a novel antenna design for chipless RFID systems at millimeter wave frequencies. The paper successfully demonstrates, in simulation, that the sophisticated antenna design based on microstrip patch antenna structure achieves a matching of about −15 dB in a frequency band of 20 GHz to 120 GHz with respect to the characteristic impedance of 50 Ohm, achieving an antenna gain of 12 dBi and an antenna efficiency of 78%.

The research paper “IKULDAS: An Improved kNN-Based UHF RFID Indoor Localization Algorithm for Directional Radiation Scenario” [7] proposes an improved UHF RFID tag localization algorithm based on the received signal strength indicator (RSSI). The paper successfully demonstrates, in theory, that the tag localization is considerably improved, taking into account the respective antenna radiation characteristics of reader and tag antennas.

The research paper “A Secure Partial RFID Ownership Transfer Protocol with Multi-Owners” [8] proposes an enhanced secure ownership transfer protocol for UHF RFID systems. The paper successfully demonstrates, in theory, a secure, high-performance multi-owner partial ownership transfer protocol that overcomes the problems of existing multi-owner tag ownership methods, addressing security threats and privacy concerns that arise in the process of ownership transfer.
